# Proteasome inhibition—a new target for brain tumours

**DOI:** 10.1038/s41420-019-0227-x

**Published:** 2019-12-04

**Authors:** Fatima Rashid, Maria Victoria Niklison-Chirou

**Affiliations:** Blizard Institute, Barts and the London School of Medicine and Dentistry, Queen Mary University of London, 4 Newark Street, London, E1 2AT UK

**Keywords:** Drug development, Paediatric cancer

The Ubiquitin Proteasome Pathway (UPP) is responsible for intracellular protein degradation in all eukaryotic cells^1^. A main function of the proteasome is to remove damaged and short-lived regulatory proteins from the cell that would otherwise accumulate and induce cell death or impair cellular function^2^. The UPP is also required to control the half-lives of regulatory ‘house-keeping’ proteins that are necessary in different concentrations depending on the cell state^2^. This highly controlled system is essential for the maintenance of protein homeostasis. As such, deregulation of the UPP has been implicated in many disease processes. Upregulation of the UPP has been linked to cancer while downregulation of the UPP has been implicated in neurodegenerative diseases such as Alzheimer’s disease^3,4^. In the context of malignancy, proteins involved in cellular differentiation, DNA damage repair, cell cycle regulation and apoptosis are targets of the UPP^5^. Many products of tumour suppressor genes and oncogenes are also subject to regulation by this pathway, and dysregulation of the UPP has been implicated in renal carcinoma, colorectal cancer, cervical cancer and glioma^4,6^. The UPP has also been shown to contribute to the neurogenesis of cerebellar progenitor cells due to its role in regulating the degradation of transcription factors involved in neuronal cell differentiation^7^. The focus of our work was to study the activity of the UPP in medulloblastoma tumours (MBs), a cancer that originates from the cerebellum^8^. Evidence from Tsvetkof et al. suggests that malignant cells are addicted to the UPP for survival^9^, and are therefore more sensitive to its inhibition than normal cells. Our recent work confirms this and shows that high proteasome activity in MB cells is associated with poor prognosis^8^. MB is the most common solid primary malignant brain tumour of children^10^. MBs have recently been classified into four molecular subgroups. These are wingless (WNT-best prognosis), sonic hedgehog (SHH-intermediate prognosis), group 3 (G3-worst prognosis) and group 4 (G4-intermediate prognosis). Aggressive medulloblastoma, G3 and G4, occurs in infants and young children and are frequently metastatic. While current treatment (surgical resection followed by chemotherapy and radiotherapy) has increased survival over the last two decades, a third of patients remain incurable and others suffer long term side effects from treatment^11^.

As cancer cells undergo proliferation at a faster rate compared to their non-malignant counterparts, they are more likely to produce proteins with synthesis errors or oxidative damage that can be cleared by an upregulated UPP, leading to cancer cell survival^12^. Unregulated proteasome activity also increases the degradation of tumour suppressors while stabilizing oncogene products^2^, leading to a shift in protein equilibrium that favours anti-apoptotic proteins such as NF-KB and BCL2. Therefore, inhibition of the UPP results in the accumulation of pro-apoptotic proteins such as p53, p73 and BAX, leading to cancer cell death^13^. The former is important in aggressive MBs because they express wild-type p53 and high levels of p73^14^. Therefore, increasing the p53 or p73 tumour suppressor function via proteasome inhibition is a feasible treatment option for p53 wild-type or p73 expressing cancers^8^. Importantly, proteasome inhibition by proteasome inhibitors (PIs) has minimal toxic effects on normal cells^15^. PIs also increase the sensitivity of tumour cells to radiotherapy and are an effective method for chemosensitization, allowing for targeted and personalized cancer treatments^16^. Furthermore, it has been reported that PIs in vivo as single agents stop tumour growth, but in combination with chemotherapy or radiotherapy induce tumour cell death^17^.

Therefore, the UPP has emerged as an attractive target for cancer treatment. PIs are drugs that block the UPP^18^. These inhibitors bind reversibly or irreversibly to the proteasome. Bortezomib was the first PI licenced for use in humans and is currently approved for use in multiple myeloma and mantle cell lymphoma as induction, and rescue treatment in relapsed/refractory disease^19,20^. Bortezomib has anti-tumour effects via p53 stabilization and NF-kB degradation followed by apoptosis^21^. Unfortunately, bortezomib has severe adverse effects such as peripheral neuropathy and neutropenia^22^. Hence, a second generation of PIs such as NPI-0052, has been developed to reduce the side effects as well as improve distribution to solid tumours. NPI-0052, also known as Marizomib, irreversibly blocks the proteasome and has demonstrated anti-tumour effects in multiple myeloma, lymphoma and glioblastoma^23,24^. Importantly, these studies also demonstrate its ability to cross the blood–brain barrier, making it a suitable treatment for brain tumours.

**Fig. 1:**
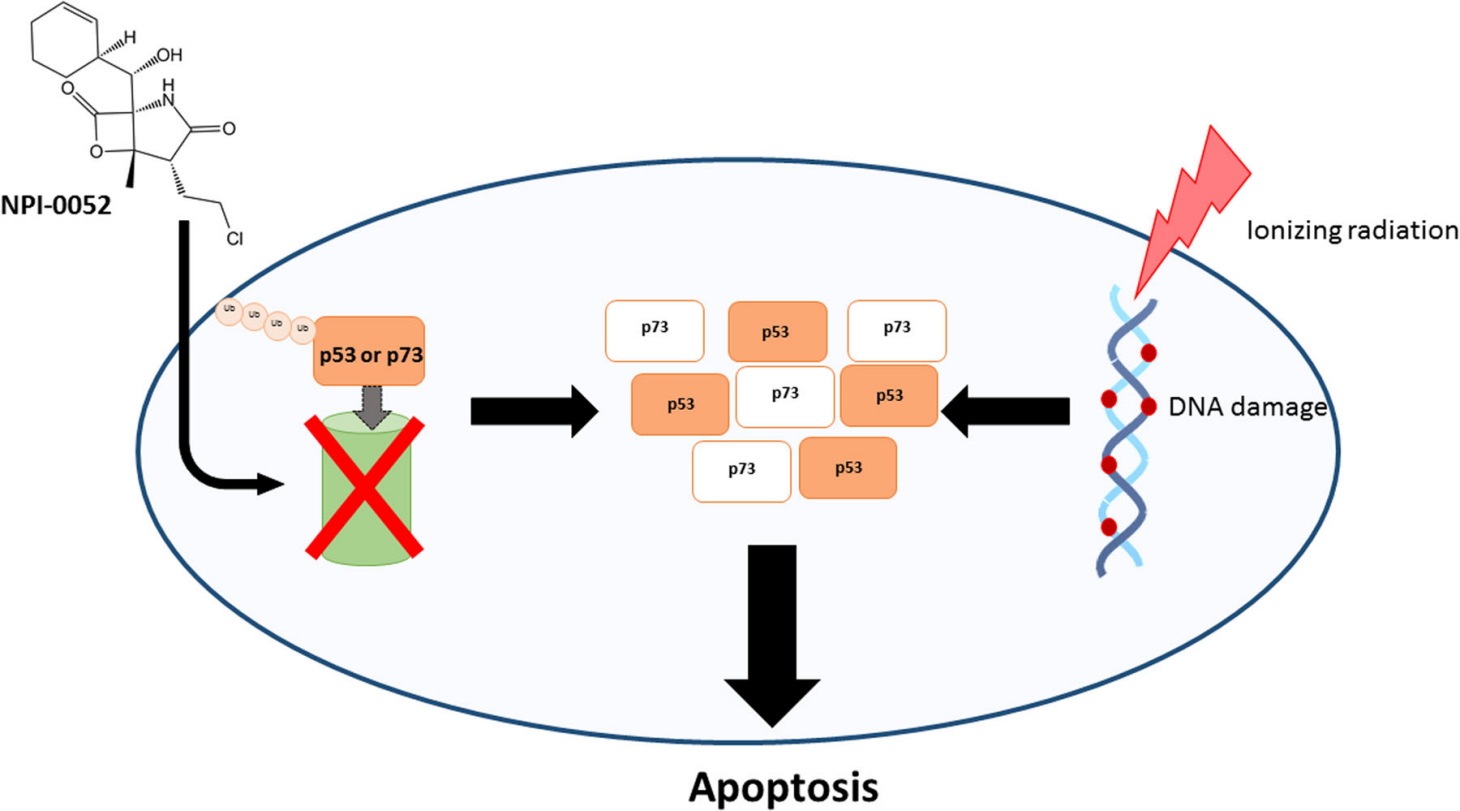
Radiosensitization of aggressive medulloblastoma cells by NPI-0052. NPI-0052 affects various growth and survival pathways in aggressive MB cells. Aggressive MBs express wild-type p53 and high levels of p73 tumour suppressor proteins. Treatment of aggressive MB with NPI-0052 will induce accumulation of p53 and p73 followed by oxidative stress in the cells. Radiation is an important component of aggressive medulloblastoma treatment. Therefore, gamma-radiation followed by NPI-0052 treatment induces DNA damage via reactive oxygen species generation, as well as p53 stabilization via proteasome inhibition. Aggressive MB cells subsequently undergo p53 mediated apoptosis.

We recently reported that NPI-0052 has anti-tumour activity in MB and induces cell death in the most aggressive types of MBs. Our findings propose a mechanism of cell death via oxidative stress, DNA damage and p53-family stabilization, adding to work undertaken by Di et al. and Miller et al.^24,25^. Importantly, we also demonstrate that gamma-radiation followed by NPI-0052 treatment results in a significant radiosensitization of aggressive MB expressing p53-family proteins (Fig. 1), a novel finding in this field. This synergistic effect was also observed in tumour organoids and provides an exciting new approach to MB treatment, with the potential of minimizing treatment related side effects.

In the 25 years since the first proteasome inhibitor was synthesised, our understanding of the molecular basis of cancers and the importance of targeted treatment has increased ten-fold. The licensing of these drugs in the treatment of haematological malignancies and now evidence of anti-cancer effects in solid tumours is a welcome and promising advancement in this era of personalized medicine.
